# Pseudoangiomatous Stromal Hyperplasia in the Breast: A Report of a Rare Case

**DOI:** 10.7759/cureus.98206

**Published:** 2025-11-30

**Authors:** Ankita Swarnkar, Madan Sundar, Kuberan Krishnan, Magesh Chandran, Ajay Gokul

**Affiliations:** 1 Medicine, Datta Meghe Institute of Medical Sciences, Wardha, IND; 2 General Surgery, Sree Balaji Medical College and Hospital, Chennai, IND; 3 General Surgery, Bharath Institute of Higher Education and Research, Chennai, IND; 4 Surgery, Bharath Institute of Higher Education and Research, Chennai, IND

**Keywords:** benign stromal growth, breast tumors, pash, proliferative breast lesion, stromal hyperplasia

## Abstract

Pseudoangiomatous stromal hyperplasia (PASH) is a benign mesenchymal proliferative breast lesion. PASH seldom forms a nodule on its own, in the absence of other breast tumours, resulting in "nodular PASH." Given its rarity, we provide a fascinating case of this poorly understood breast pathology in a 25-year-old woman who was first diagnosed with fibroadenoma of the left breast. Breast ultrasonography and mammography are the most commonly used procedures for evaluating breast diseases. Fibroadenomas can have the same clinical and radiological presentation as nodular PASH. Because of the non-specific features of nodular PASH, histopathological testing remains the gold standard for confirming this unusual diagnosis and ruling out low-grade angiosarcoma. Patients with nodular PASH have a great prognosis following excision. PASH is a benign stromal growth that histologically induces a vascular lesion; additional instances must be reported in order to generate thorough guidelines for managing it.

## Introduction

Pseudoangiomatous stromal hyperplasia (PASH) is a benign mesenchymal proliferative breast lesion first identified by Vuitch et al. in 1986 [1]. It is distinguished by a dense network of slit-like gaps within the breast stroma that resemble vascular channels but lack endothelial lining. PASH is a type of benign stromal proliferation of the breast that is hypothesised to be caused by myofibroblasts.

This condition primarily affects premenopausal and menopausal women on hormone replacement treatment (HRT) [2]. There were very few occurrences of PASH in men with gynaecomastia [3]. The specific pathophysiology of PASH is unknown, although due to its frequency in premenopausal women, it is considered hormonally driven [4].

Clinically, PASH can show in various ways, ranging from an incidental histologic discovery to a clinically identifiable mass [5]. Up to 23% of consecutive breast samples may have accidental microscopic PASH [6]. However, it is uncommon for PASH to develop a nodule without accompanying lesions, resulting in "nodular PASH" [7]. The incidence of nodular PASH might be as low as 1-2% [8].

Fibroadenoma is a clinical and imaging mimicker of nodular PASH, which explains why many nodular PASH cases are misdiagnosed as breast fibroadenoma [9]. The literature describes approximately 150 cases of PASH. Given its rarity, we provide an intriguing case of this poorly understood breast disease in a 53-year-old woman who was first diagnosed with fibroadenoma of the left breast.

Surgery is frequently advised for people with nodular PASH, particularly if they are symptomatic or have a significant history of breast cancer. However, if PASH is histologically confirmed and there are no suspicious radiologic signs or a history of breast cancer, regular follow-ups and imaging can be explored, especially if the patient is asymptomatic. PASH has a great prognosis, with a recurrence risk of up to 21%, particularly if no thorough excision was performed.

## Case presentation

A 53-year-old multiparous woman presented to our hospital in January 2025 with a long-standing history of a lump in both breasts since 1998, which had been insidious in onset and non-progressive in nature. She also complained of intermittent bilateral breast pain and a greenish, serous, non-foul-smelling nipple discharge that had become more noticeable over the past few months. Her past medical and surgical history was uneventful, with no known drug or food allergies. She had regular menstrual cycles, had never used hormonal contraceptives, and reported no family history of breast or ovarian malignancy.

On clinical examination, both breasts were tender on palpation, and mild fullness was noted in the upper outer quadrants. The nipple-areolar complexes appeared normal, with greenish discharge expressible from multiple ducts bilaterally. No discrete mass, nipple retraction, peau d’orange, or skin thickening was observed. Both axillae were mildly tender but without palpable lymphadenopathy, and there was no supraclavicular node enlargement.

**Figure 1:**
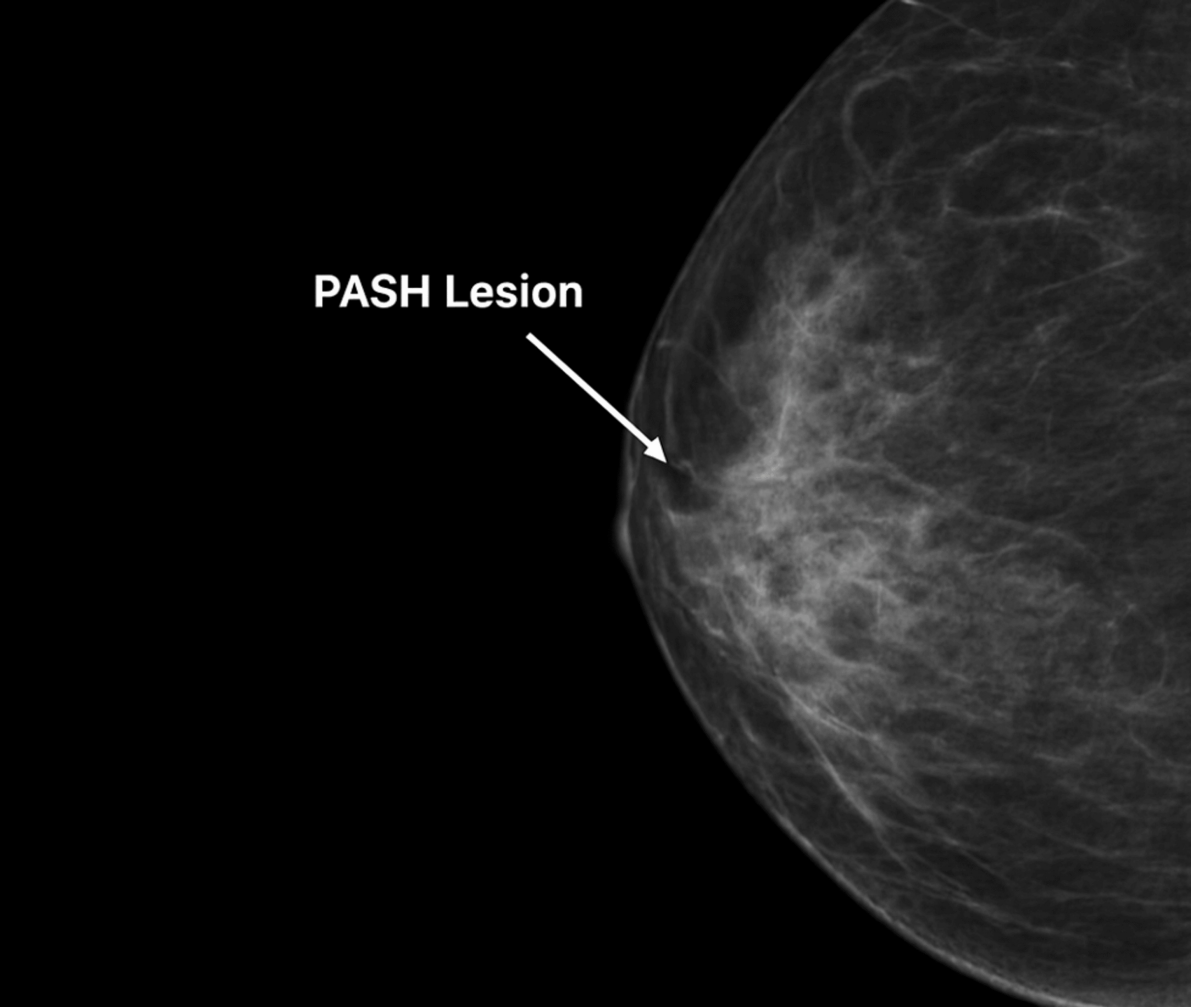
Mammogram showing moderately dense breasts with a dilated tubular structure in the retroareolar region. In addition, a well-circumscribed, dense opacity with smooth margins, suggestive of PASH, is seen.

**Figure 2:**
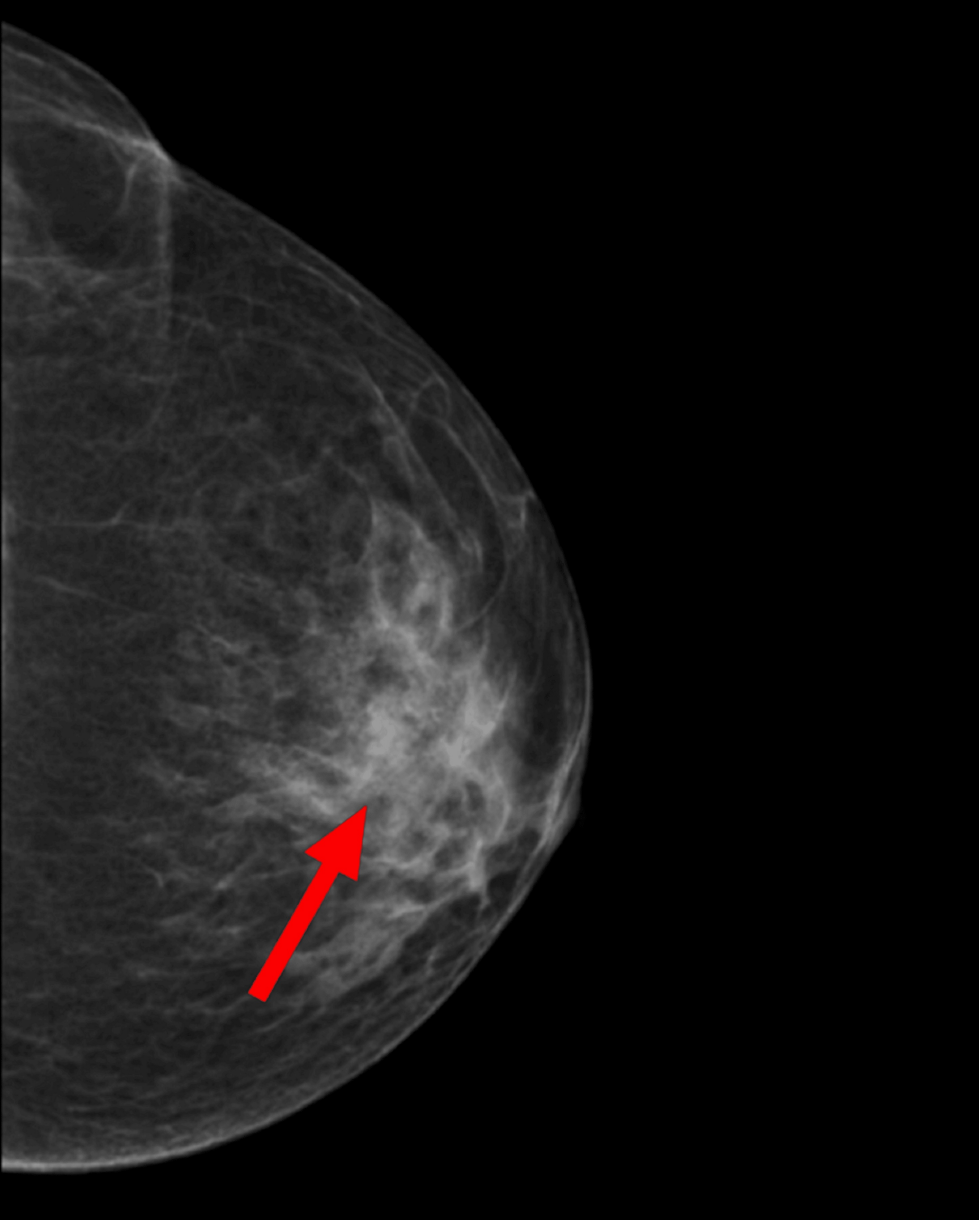
Mammogram showing a dense, circumscribed, opaque nodule (red arrow) in a mediolateral oblique view of the left breast.

**Figure 3:**
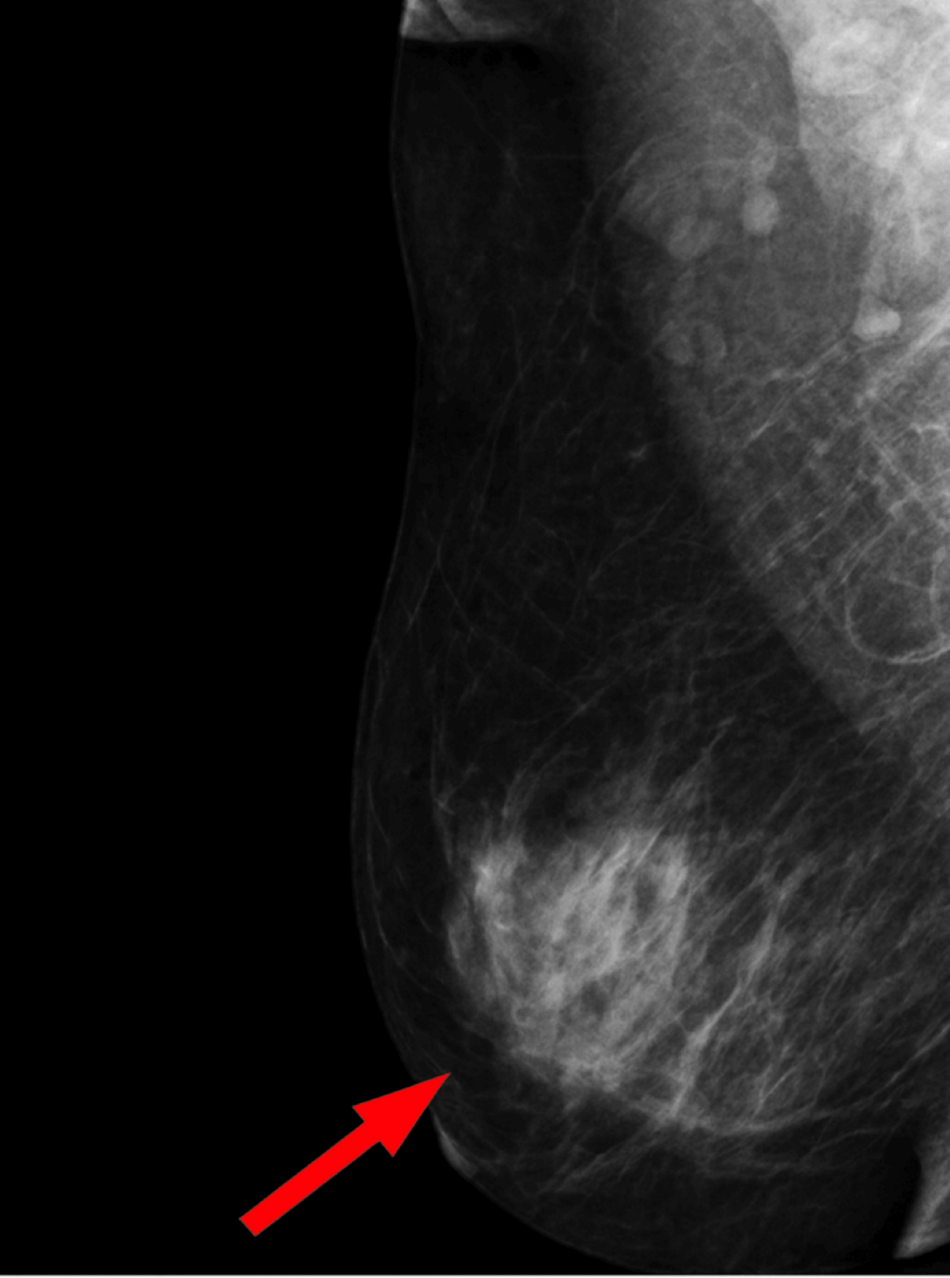
Mediolateral oblique (MLO) mammogram of the left breast showing a focal dense stromal area (marked) corresponding to biopsy-proven pseudoangiomatous stromal hyperplasia (PASH).

**Figure 4:**
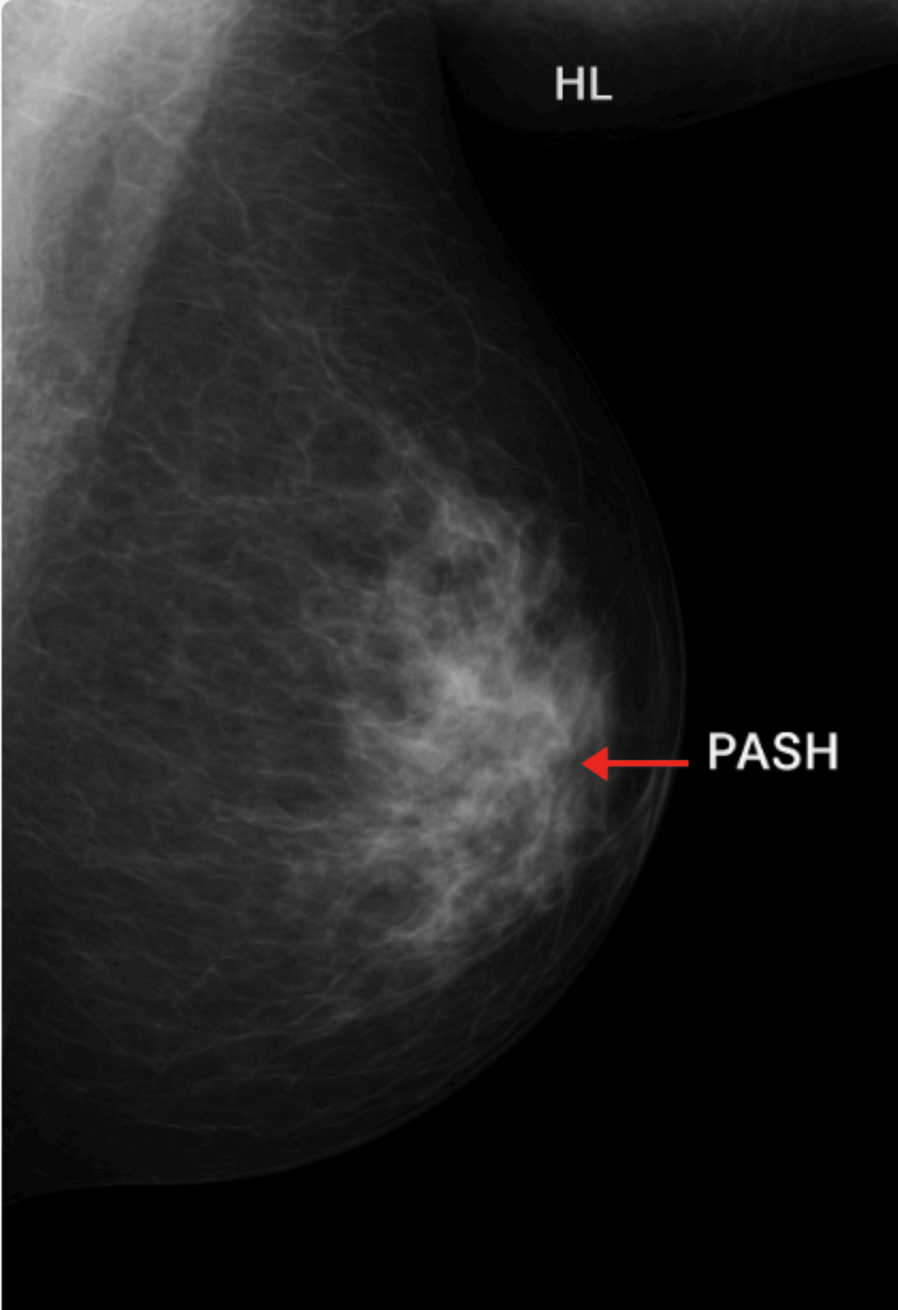
Mediolateral oblique (MLO) view of the left breast demonstrating a focal area of increased fibroglandular density in the retroareolar to lower-inner quadrant, indicated by the arrow. This region corresponds to the site of biopsy-proven pseudoangiomatous stromal hyperplasia (PASH). The lesion appears as an ill-defined, heterogeneous density without associated calcifications or architectural distortion, a typical presentation of PASH on screening mammography.

Bilateral mammography revealed multiple dilated ducts measuring approximately 2.3 mm at the 10-11 o'clock position in the right breast and 2.7 mm at the 12-1 o'clock position in the left breast. A few subcentimetric axillary lymph nodes with preserved fatty hila were seen on both sides. The findings were classified as Breast Imaging Reporting and Data System (BI-RADS) Category 2, consistent with benign ductal dilatation (Figures 1-4).

**Figure 5:**
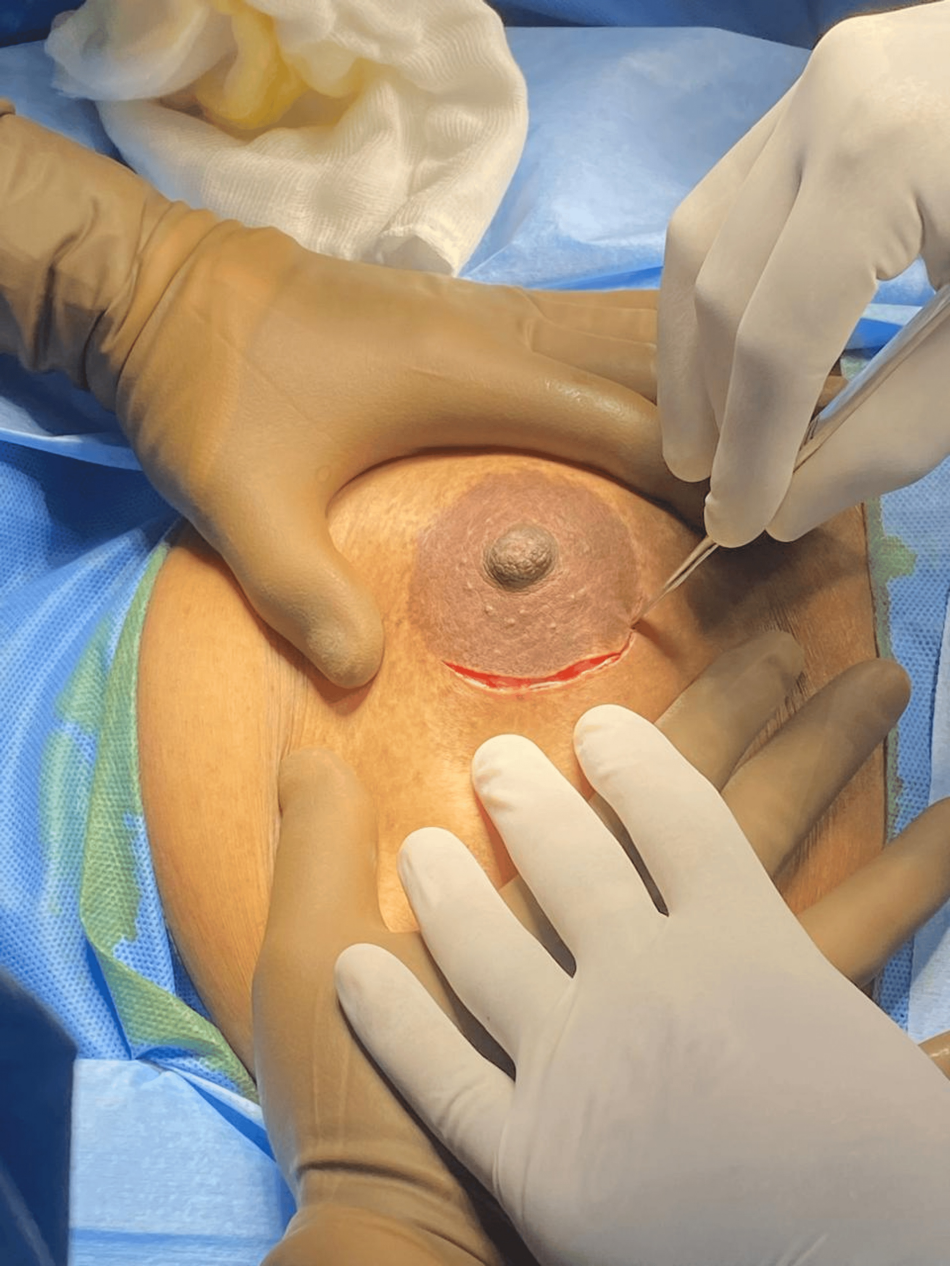
Intraoperative field.

Considering the patient's persistent symptoms and documented ductal enlargement, the surgical team recommended bilateral microdochectomy both for definitive diagnosis and symptom relief. After informed consent, the procedure was performed under epidural anaesthesia. Through a circumareolar incision, the involved ducts were identified by gentle probing, dissected free from surrounding parenchyma, ligated, and excised at the 10-11 o'clock position in the right breast and the 12-1 o'clock position in the left breast. The excised ducts were submitted for histopathological examination (Figure 5).

**Figure 6:**
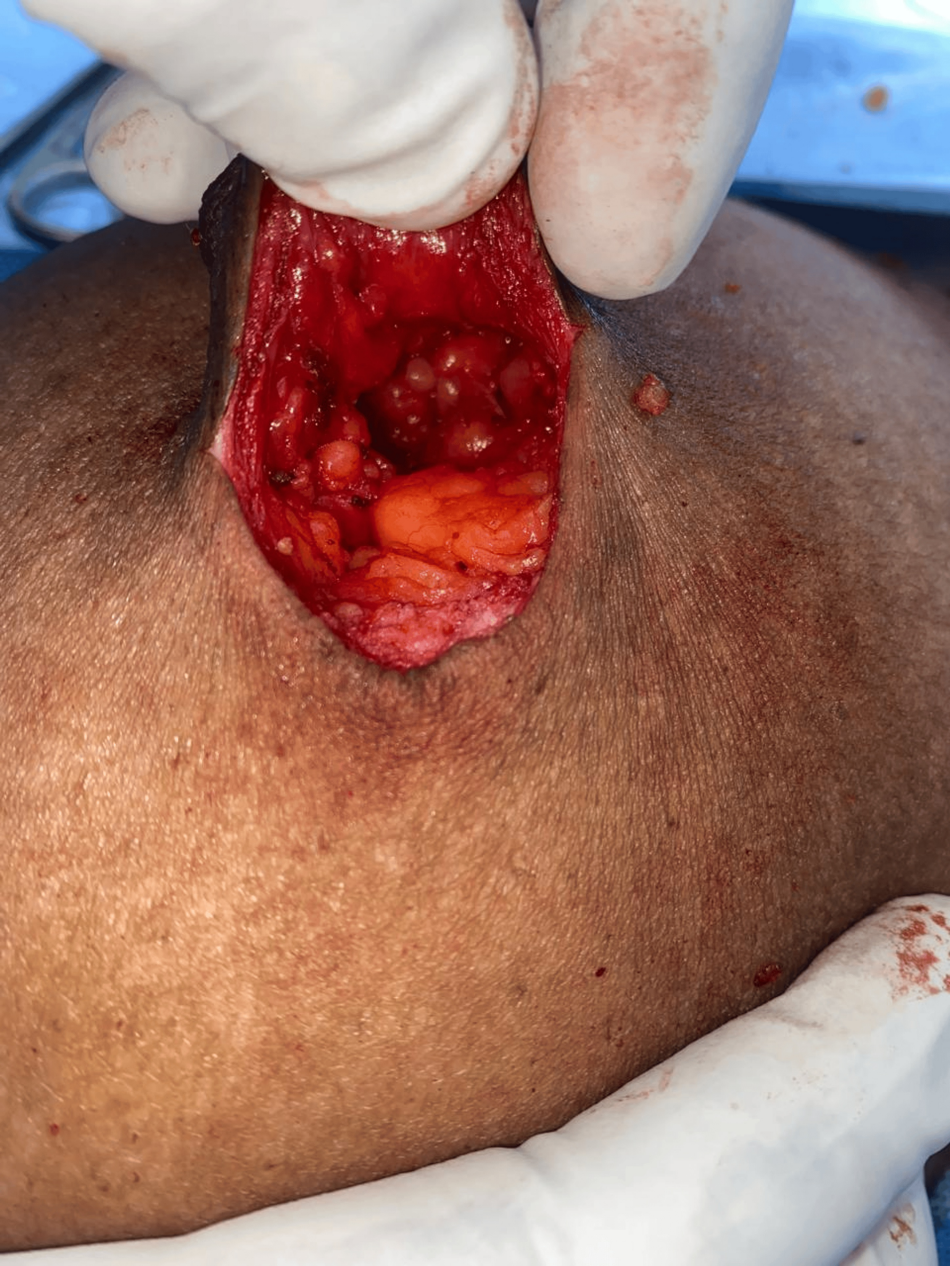
The intra-operative field showing exposed fibro-glandular tissue.

**Figure 7:**
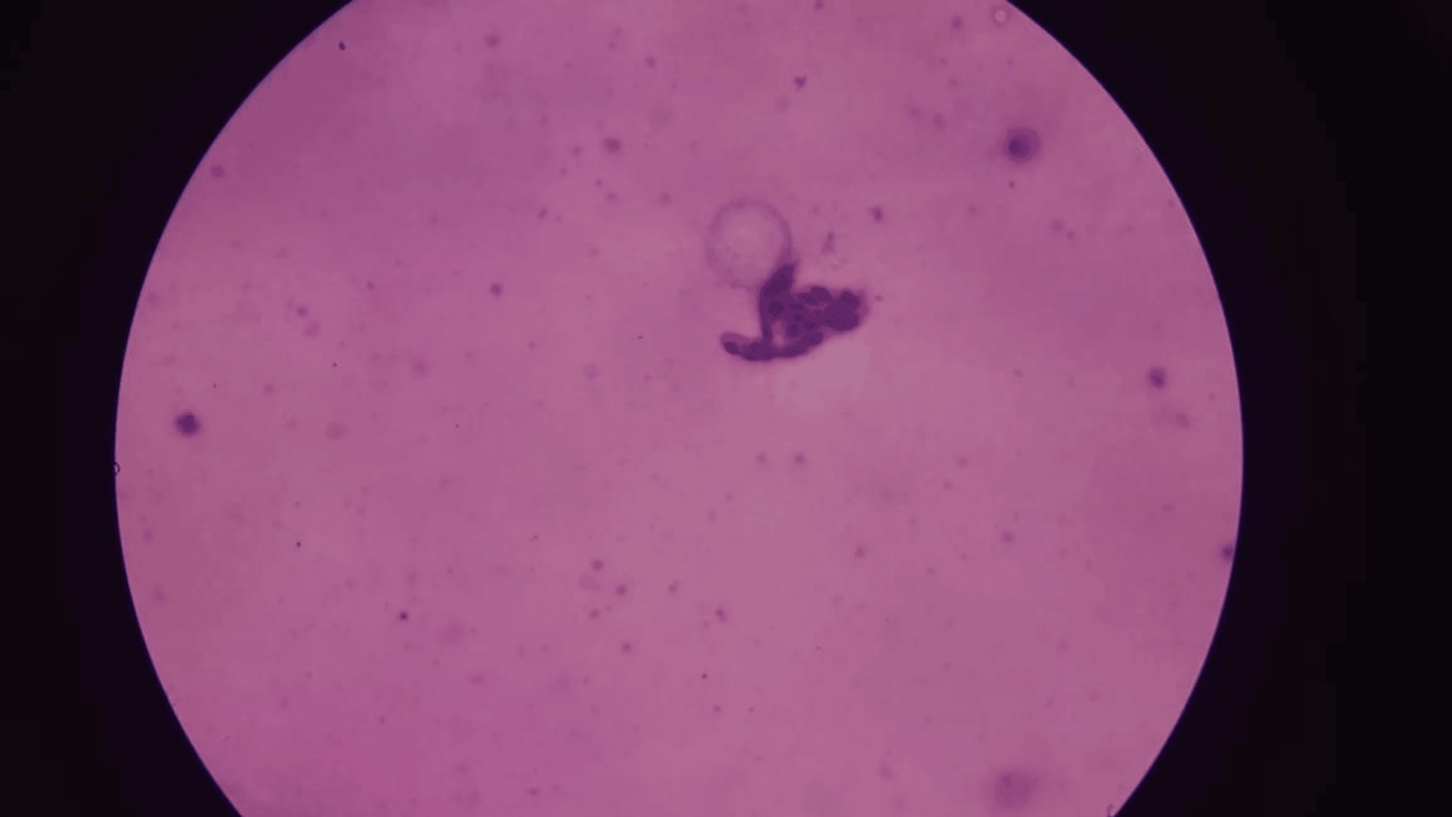
Cytology showing duct ectasia with dilated ducts and periductal inflammation, along with slit-like stromal spaces lined with benign spindle cells.

Microscopic evaluation of the specimens demonstrated markedly dilated ducts lined by flattened to cuboidal epithelium with dense periductal fibrosis and chronic inflammatory infiltrate composed predominantly of lymphocytes and plasma cells. Focal apocrine metaplasia and cystic change were present, without epithelial atypia, necrosis, or invasive components. These findings confirmed the diagnosis of bilateral duct ectasia with associated fibrocystic changes (Figures 6, 7).

Following histopathological confirmation of the benign nature of the disease, the patient was counselled and reassured. Postoperative management included a five-day course of oral amoxicillin-clavulanate for infection prophylaxis and non-steroidal anti-inflammatory drugs for analgesia. She was advised to avoid nipple manipulation, maintain good breast support with a firm brassiere, and return for scheduled follow-up visits. At the two-week review, wound healing was satisfactory with no evidence of seroma or discharge. At three months, the patient reported complete resolution of pain and absence of nipple discharge. At six months, clinical examination and ultrasonography of both breasts were normal with no signs of recurrence.

This case thus illustrates a rare presentation of bilateral duct ectasia with fibrocystic changes, effectively managed by microdochectomy and conservative postoperative care, resulting in complete symptomatic relief and an excellent short-term outcome.

## Discussion

PASH is a benign stromal growth that histologically resembles a vascular lesion [10]. It might appear with a variety of clinical symptoms, making diagnosis difficult. While it most commonly affects women in their 30s and 40s, it has also been documented in paediatric and postmenopausal patients, particularly those using HRT.

The lesion's development patterns and ability to mimic other breast diseases underline the need for clinical awareness and suitable therapeutic measures [4]. Our case involved a 53-year-old woman with a history of a breast lump over a period of more than 20 years. As a result, clinical suspicion of PASH should be assessed in conjunction with the patient's menopausal status.

Kurt et al. found that the majority of patients had tumours measuring less than 2 cm [11]. In contrast, we described a lump that was non-progressive in nature.

The most frequent kind of PASH is tiny and typically asymptomatic. According to certain research, up to 23% of premenopausal women can have microscopic PASH on pathologic examination, which is frequently associated with other breast diseases [6]. On the other hand, PASH, as the major pathologic finding or "nodular PASH," responsible for a single nodule, is uncommon.

There have been very few published occurrences of PASH in males with gynaecomastia [3]. Breast ultrasound and mammography are the most common procedures for evaluating breast diseases [12]. Given our patient's age, mammography was used to assess her breast nodule.

The sonographic features of PASH are often vague. PASH usually appears as a benign, hypoechoic, oval-shaped, well-defined tumour [13]. These identical characteristics were not observed in our case.

On mammography, PASH is typically seen as a non-calcified, spherical, well-circumscribed mass or a focal asymmetry [13]. T1-weighted gradient-echo images on magnetic resonance imaging often indicate an isointense mass. It may exhibit a straight reticular "lacelike" pattern on axial T2-weighted imaging, indicating the presence of slit-like gaps inside the lesion [5].

Given the nonspecific radiologic features of PASH, histopathological testing remains the gold standard for establishing the unusual diagnosis and ruling out malignancy.

PASH pathology often reveals a spherical, rubbery, well-circumscribed tumour, similar to the one we isolated in our instance. Cysts may occasionally appear [14]. On microscopy, PASH appears as a complex arrangement of slit-like gaps lined by endothelial-like spindle cells surrounded by dense collagenous stroma.

**Figure 8:**
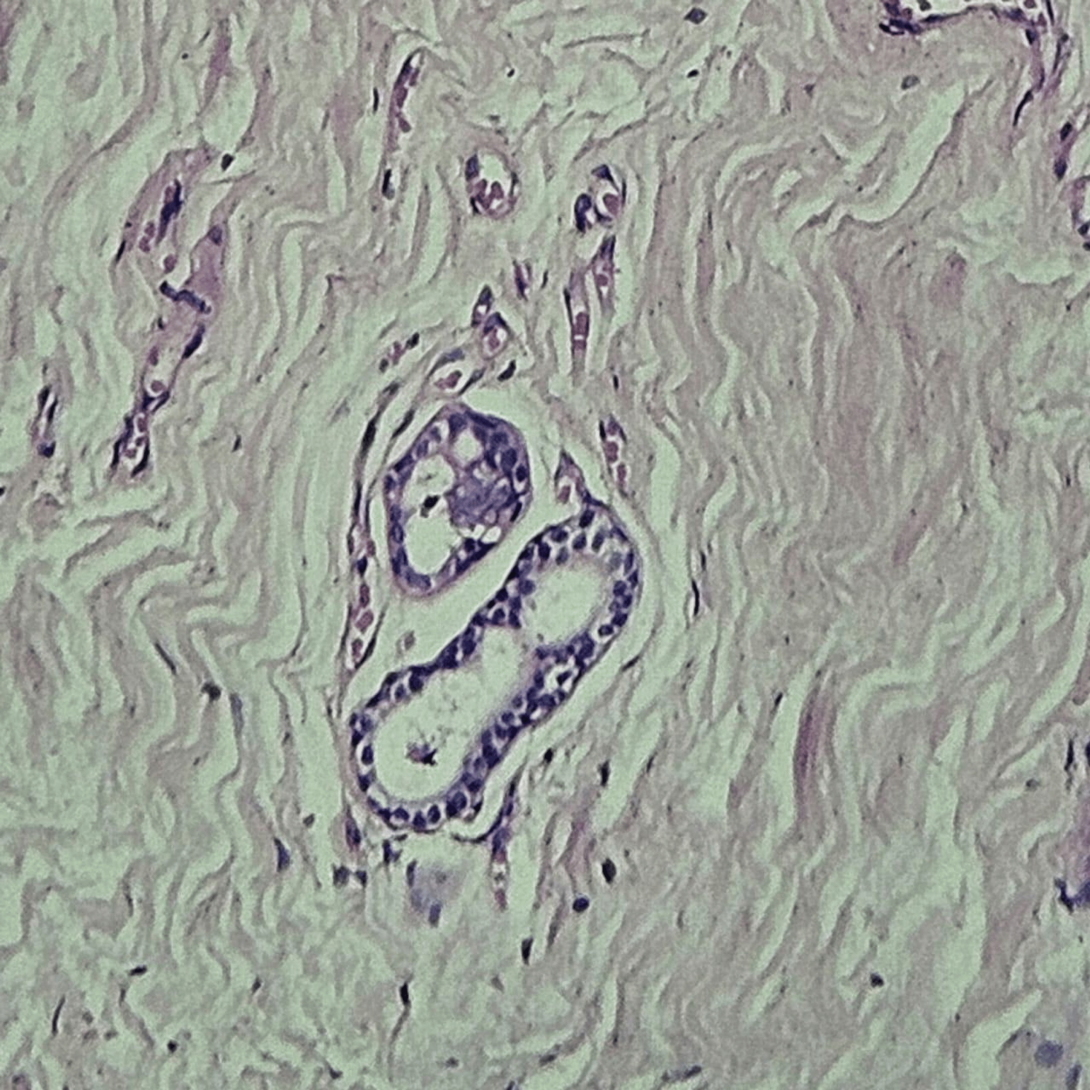
Nipple histology showing benign ductal epithelial cells with foamy macrophages and inflammatory cells, consistent with duct ectasia.

The proliferation of fibroblasts and myofibroblasts, along with excessive collagen production, results in solid tissue interspersed with cystic patches that resemble dilated arteries. These stromal cells are hypothesised to respond excessively to progesterone. These usual results are consistent with our microscopic observations (Figure 8).

Other histologic findings may be noted, as PASH exhibits a wide range of histopathologic characteristics. PASH's differential diagnosis includes fibroadenoma, myofibroblastoma, mammary hamartoma, and angiosarcoma. Angiosarcoma is the most important differential diagnosis to rule out due to its malignant character.

Angiosarcoma appears as an infiltrative, haemorrhagic tumour with irregular boundaries. Microscopic analysis of PASH and angiosarcoma reveals anastomosing gaps that are lined with spindle cells. However, discrepancies in the findings become more visible at various magnifications. In low-power microscopy, angiosarcoma destroys the interlobular stroma and invades the surrounding fat, whereas PASH gradually merges with the nearby stroma.

At high magnification, angiosarcoma gaps are lined by atypical endothelial cells with mild to moderate pleomorphism, nuclear atypia, and enhanced mitosis. These cells range in size and form, with hyperchromatic nuclei. These areas may also contain haemorrhage with red blood cells. In contrast, the spindle cells observed in PASH are benign and exhibit no cytologic atypia or mitotic activity.

The histologic characteristics in our patient were consistent with PASH, with no indication of cytologic atypia or invasive growth. A careful review and multidisciplinary discussion with our pathology team found that immunostaining was unnecessary because the diagnosis was clear based on appearance alone. Histological investigation revealed that the lesion was benign, with no cytologic atypia, mitotic activity, or invasive characteristics.

PASH can coexist with malignant breast diseases such as ductal carcinoma in situ and invasive ductal carcinoma [15], which are characterised by unusual radiologic features. Immunohistochemical staining helps to distinguish between the two situations. Spindle cells in angiosarcoma express endothelium markers such as CD31, CD34, and the von Willebrand factor antigen. PASH spindle cells express myofibroblastic markers such as CD34 and SMA but lack endothelial markers [16]. These lesions often have a good prognosis, although recurrence rates are higher if the lesion is not entirely excised [16]. In terms of treatment, breast microdochectomy is typically indicated for symptomatic individuals with nodular PASH, those at high risk for breast cancer, and BI-RADS 2. This supports the need for regular follow-ups.

Patients with a histologic diagnosis of PASH, no worrisome radiologic abnormalities, and no history of breast cancer may benefit from regular imaging and follow-up, particularly if they are asymptomatic [17]. Fortunately, PASH does not enhance the risk of cancer.

Finally, if individuals with growing or painful breast lumps refuse surgery, medicinal management may be an alternative. Tamoxifen, a selective oestrogen receptor modulator, has been shown to alleviate similar symptoms. However, because of its anti-oestrogen side effects, tamoxifen is often used for short periods of time [18].

## Conclusions

PASH is a benign stromal growth that histologically resembles a vascular disease. It is a rare occurrence, particularly of the nodal form. It is frequently seen in premenopausal or postmenopausal women on HRT and is assumed to be hormonally influenced. It can mimic various breast diseases, and it is critical to distinguish it from low-grade angiosarcoma. Immunohistochemistry may be beneficial. Patients with PASH have a very good prognosis after excision. Finally, considering the rarity of PASH, reporting more cases is critical for developing thorough management guidelines.
